# Comparison of Cas12a and Cas9-mediated mutagenesis in tomato cells

**DOI:** 10.1038/s41598-024-55088-4

**Published:** 2024-02-24

**Authors:** Ellen Slaman, Lisanne Kottenhagen, William de Martines, Gerco C. Angenent, Ruud A. de Maagd

**Affiliations:** 1 Laboratory of Molecular Biology, Wageningen University & Research, Wageningen, The Netherlands; 2 Bioscience, Wageningen University & Research, Wageningen, The Netherlands

**Keywords:** Plant biotechnology, Plant sciences

## Abstract

Cas12a is a promising addition to the CRISPR toolbox, offering versatility due to its TTTV-protospacer adjacent motif (PAM) and the fact that it induces double-stranded breaks (DSBs) with single-stranded overhangs. We characterized Cas12a-mediated genome editing in tomato using high-throughput amplicon sequencing on protoplasts. Of the three tested variants, *Lachnospiraceae* (Lb) Cas12a was the most efficient. Additionally, we developed an easy and effective Golden-Gate-based system for crRNA cloning. We compared LbCas12a to SpCas9 by investigating on-target efficacy and specificity at 35 overlapping target sites and 57 (LbCas12a) or 100 (SpCas9) predicted off-target sites. We found LbCas12a an efficient, robust addition to SpCas9, with similar overall though target-dependent efficiencies. LbCas12a induced more and larger deletions than SpCas9, which can be advantageous for specific genome editing applications. Off-target activity for LbCas12a was found at 10 out of 57 investigated sites. One or two mismatches were present distal from the PAM in all cases. We conclude that Cas12a-mediated genome editing is generally precise as long as such off-target sites can be avoided. In conclusion, we have determined the mutation pattern and efficacy of Cas12a-mediated CRISPR mutagenesis in tomato and developed a cloning system for the routine application of Cas12a for tomato genome editing.

## Introduction

The application of the CRISPR-Cas9 system in plants has enabled targeted mutagenesis with unprecedented speed and simplicity^1–6^. However, the number of mutable genomic targets is limited by the requirement for an “NGG” protospacer adjacent motif (PAM) for cleavage. A promising addition to the CRISPR toolbox is the Cas12a nuclease, which differs from Cas9 in several aspects^7^. First, it requires a 5’ “TTTV” PAM instead of the 3′ “NGG” of Cas9. The alternative PAM may make it easier to find Cas12a target sites than Cas9 sites in A/T-rich genomic regions, such as promoters. Additionally, Cas12a induces double-stranded breaks (DSBs) with a 4–5 bp overhang, in contrast to the blunt breaks induced by Cas9. These overhangs (“sticky ends”) may prove helpful in targeted integration approaches. Recently, they were shown to be advantageous for achieving precise integrations in the genomes of mammalian cells through a combined mechanism of microhomology-mediated end joining (MMEJ) and homology-directed repair (HDR)^8^. Furthermore, Cas12a CRISPR-RNAs (crRNAs) only need a short, 21–36 bp direct repeat 5’ of the spacer to provide the correct structure to the crRNA for proper loading in the nuclease. Cas9 needs two RNAs: a crRNA and a transactivating-crRNA, often combined in a single guide RNA (sgRNA) with a combined length of ~ 100 bp^9^. Finally, Cas12a is also a ribonuclease able to process its CRISPR arrays, allowing the application of such arrays for multiplexing^10,11^.

Cas12a variants from *Acidaminococcus, Francisella novicida,* and *Lachnospiraceae* (AsCas12a, FnCas12a, and LbCas12a, respectively) were shown to reliably induce mutations in mammalian cell lines, and Cas12a was quickly adopted as an efficient genome editing tool^12–14^. As an added benefit, Cas12a seemed to induce fewer off-target mutations than Cas9^15,16^. In plants, however, the nuclease was less readily applied. Early reports of the application of Cas12a for rice genome editing – the first plant species reported to be edited using Cas12a—revealed low editing efficiencies^17,18^. Editing efficiencies were subsequently improved by using specific methods for crRNA expression and increasing Cas12a nuclease activity^19–21^.

Tomato is an economically important crop, as well as a model species for research on fleshy fruits. Cas9-mediated mutagenesis has been readily and frequently applied to tomato and was used to study, among other traits, plant architecture, fruit development, and (a)biotic stress tolerance^22,23^. However, only a few reports using Cas12a have been published^24–26^. The slow and limited adoption of Cas12a might be due to limited data on the performance of Cas12a in the tomato genome and the absence of an efficient, easy-to-use cloning system for crRNA expression.

The components needed for CRISPR-Cas mutagenesis in tomato are often delivered to the plant through *Agrobacterium tumefaciens-*mediated transformation^27^. Although effective, regenerating stably transformed plants through tissue culture is laborious and, therefore, not particularly suitable for the optimization of CRISPR-mediated genome editing techniques. Consequently, we focused our efforts on protoplasts. Previously, we developed a method for 96 well-format protoplast transfections and coupled this to next-generation amplicon sequencing to study the characteristics and specificity of CRISPR-Cas9-mediated genome editing in tomato^28^. In this work, we used a similar approach to compare multiplex crRNA expression strategies and developed an efficient, easy-to-use Golden Gate-based system for crRNA expression. Additionally, we compared Cas12a and Cas9 for efficacy, mutational pattern, and specificity on a set of overlapping targets. To achieve this, we selected 35 overlapping target sites for Cas9 and Cas12a in the *bHLH* transcription factor gene family and determined on- and off-target mutations for the corresponding crRNAs and sgRNAs. We found Cas12a a reliable and robust addition to Cas9 genome editing. Additionally, our study revealed that Cas12a preferentially induces more and larger deletions than Cas9—a trait that may be useful when specific mutational outcomes are desired. These data pave the way for the routine application of Cas12a in mutagenesis experiments in tomato.

## Materials & methods

### Selecting target sites and off-target sites

For the initial Cas12a optimization experiments, CRISPOR^29^ was used to identify Cas12a target sites in the first exons of the tomato *PHYTOENE DESATURASE (PDS)* gene (*Solyc03g123760*).

To identify overlapping target sites in transcription factor gene families, coordinates from all exons that are part of coding sequences were extracted from the ITAG4.0_gene_models.gff file, obtained from solgenomics.net (grep -w "CDS" ITAG4.0_gene_models.gff). The resulting file was converted to a BED-file, and corresponding DNA sequences were extracted from the ITAG4.0 tomato genome build using BEDtools. Using a list of transcription factors obtained from the Plant Transcription Factor Database^30^ and a regular expression describing the target sites, overlapping target sites were identified in coding sequences for transcription factors using a Python script. All off-target sites for the identified target sites with a maximum of 3 mismatches and a maximum of one-nucleotide DNA/RNA bulge were predicted using CasOFF-Finder^31^, for both enzymes. Thirty-five target sites in the *bHLH* gene family with predicted off-target sites for both Cas12a and Cas9 were selected for further testing. Primers for amplifying on-target sequences and a selection of predicted off-target sequences were designed using BatchPrimer3^32^ in DNA sequences surrounding the target and predicted off-target sites extracted from the ITAG4.0 tomato genome build using BEDtools.

### Vector construction

All vector assembly was done with Golden Gate cloning, using parts from the MoClo toolkit^33^ (Addgene #1000000044) and from the MoClo Plant Parts kit^34^ (Addgene #1000000047), unless otherwise described. Used primer sequences can be found in Supplementary Dataset 3. Schematic overviews of the cloned plasmids can be found in Supplementary Fig. 1.

Plasmids encoding human codon-optimized AsCas12a, FnCas12a, and LbCas12a containing a nuclear localization signal and 3xHA tag at the 3’end were gifts from the Feng Zhang lab^7^. They were obtained through Addgene (accession numbers 69982, 69988, and 69976, respectively). The nuclease genes were amplified with primers adding flanking *Bpi*I sites (Supplementary Dataset 3) and subsequently inserted in the level 0 vector for coding sequences, pICH41308, using restriction-ligation. The coding sequences were then combined with the CaMV35S promoter and NOS terminator (pICH51288 and pICH41421, respectively) in pICH47742.

To create vector backbones for crRNA expression, a Cas9-based CRISPR-Pink cassette was used as a basis (a gift from Marc Youles, The Sainsbury Laboratory). The AtU6-26 promoter was amplified using a reverse primer, adding the direct repeat sequence for either AsCas12a, FnCas12a, or LbCas12a, with either the mature or the pre-crRNA sequence and a flanking *Bsa*I site introducing an overhang to allow seamless cloning to the CRISPR-Pink RFP operon. This RFP was then amplified with primers, adding *Bsa*I sites with compatible overhangs to fuse this part to the AtU6-26 promoter with a direct repeat sequence. The two amplicons were then combined into level 1, position 1 to 7 backbone vectors (pICH47732, pICH47742, pICH47751, pICH47761, pICH47772, pICH47781, and pICH47791) using restriction-ligation to create the final crRNA expression cassettes. Primer sequences can be found in Supplementary Dataset 3.

For our initial crRNA expression optimization experiments, we selected three target sites in *SlPDS*, designed and annealed oligonucleotides (Supplementary Dataset 2), and ligated these into the previously constructed crRNA expression cassettes, following the protocol as described in Supplementary Information 2. These crRNA expression vectors were then combined with pICSL7004 (*NPTII*), the constructed *AsCas12a*, *FnCas12a,* or *LbCas12a* expression vector, a *tGFP* marker (a combination of pICH41414, pICH51288, and pICH41414 in pICH47751) and end-linker pICH41822 in pICSL4723 to form binary multiplexing level 2 vectors. Additionally, arrays encoding the three selected crRNAs each transcribed from their own AtU6-26 promoter in both pre-crRNA form and mature form, were synthesized for all nucleases (GenScript, sequences can be found in Supplementary Information 1). These arrays were subsequently cloned to a level 1, position 4 backbone (pICH47761) and again combined with pICSL7004, the nuclease, a *tGFP* marker, and end linker pICH41780 into pICSL4723 to form level 2 binary vectors.

To clone the vector expressing the mature LbCas12a crRNA array using a PolII promoter, the array was amplified with primers, adding overhangs to allow cloning into a level 0 vector for coding sequences (pICH41308). The array was subsequently combined with a Cassava Vein Mosaic Virus (CsVMV) promoter (pICSL12006) and Mannopin Synthase (MAS) terminator (pICH77901) into a level 1, position 4 backbone (pICH47761). The crRNA expression cassette was combined with NPTII, LbCas12a and tGFP into a binary level 2 vector as described above.

For the expression system using ribozymes, the LbCas12a crRNA array was amplified and subsequently cloned in pGEM-T Easy (Promega), according to the manufacturer’s instructions. This allowed it to function as a level -1 part. The Hepatitis Delta Virus (HDV) and Hammerhead (HH) ribozymes were amplified from Addgene plasmid #86197, which was a gift from Tang et al^19^., and similarly cloned to pGEM-T Easy (Promega). The three parts were subsequently combined in a level 0 vector for coding sequences (pICH41308), combined with the CsVMV promoter and MAS terminator, and next combined with *NPTII*, *LbCas12a,* and *tGFP* as described above.

For later experiments with the 35 overlapping target sites, we constructed level 2 backbones in which a single crRNA or sgRNA could easily be inserted. A schematic overview can be found in Supplementary Fig. 1c. For the LbCas12a variant, a thermotolerant, Arabidopsis codon-optimized LbCas12a was used, which was a gift from the Puchta lab^20^. Modifications were made to this ttLbCas12a to include two 5’ SV40 nuclear localization signals. A potato IV2 intron was added after the second NLS to prevent bacterial expression of the Cas12a protein. Additionally, a 3’ nucleoplasmin NLS and a third SV40 NLS were added. This modified ttLbCas12a was combined with a CaMV35S promoter (pICH51288) and NOS terminator (pICH41414) into pICH47742. For SpCas9, the same two 5’ SV40 NLS with the potato IV20 intron were added, and the nuclease was then combined with a CaMV35S promoter and NOS terminator into pICH47742. To be able to clone crRNAs directly in binary level 2 vectors, the *Bsm*BI sites in both the level 1, position 6 CRISPR-Pink backbones for Cas9 sgRNA and LbCas12a mature crRNA expression were replaced by *Bsa*I sites. In both these CRISPR-Pink backbones, crRNAs or sgRNAs are expressed using the *AtU6-26* promoter. For the final vectors, *NPTII* (pICSL7004) was combined with either the modified *ttLbCas12a* or *SpCas9*, *tGFP*, pICH54055, pICH54066, the *Bsa*I-adapted CRISPR-Pink vector for crRNA or sgRNA expression, and end-linker pICH41822 into pICSL4723. The 35 sgRNAs and crRNAs were subsequently cloned into their respective backbones by introducing the spacer, as annealed oligonucleotides, in the CRISPR-Pink module by restriction/ligation using *Bsa*I. Sequences of the oligonucleotides can be found in Supplementary Dataset 2.

### DNA preparation

Highly pure DNA for transfection was prepared from 3 mL of overnight *E. coli* culture in LB medium using the PureYield Plasmid MiniPrep System (Promega), with the following adaptations^28^: bacterial pellets were frozen at − 20 °C before processing to increase DNA yield, the column was washed twice with the endotoxin removal wash to acquire the desired purity, and plasmid DNA was eluted with 30 uL elution buffer preheated at 60 °C.

### Protoplast isolation and transfection

Protoplast isolation and transfection in 96-well format were performed as described in ^28^.

### Genomic DNA isolation and amplicon sequencing

Protoplast DNA was purified from entire protoplast pools 24 h after transfection using magnetic beads (NucleoMag Plant, Macherey–Nagel), following the manufacturer’s instructions. DNA was eluted in 50 µL, of which 6 µL was subsequently used as a template in 25 µL PCR reactions using PHUSION HotStart Flex DNA polymerase (NEB) to amplify genomic DNA fragments containing target or predicted off-target sites using barcoded primers. For the PCR, an initial denaturation for 30 s at 98 °C was followed by 38 cycles of denaturation for 10 s at 98 °C, annealing for 20 s at 58 °C, extension for 20 s at 72 °C, and a final extension step of 3 min at 72 °C. Primer sequences are listed in Supplementary Dataset 3. The resulting PCR products were visualized by electrophoresis on a 2% agarose gel. Equal amounts of PCR products were pooled to obtain sequencing libraries. Libraries were subsequently column-purified with the NucleoSpin Gel and PCR Clean-up kit (Macherey–Nagel), following the manufacturer’s instructions. Illumina HiSeq sequencing (paired-end, 2 × 150 bp reads) was performed by Eurofins Genomics Europe Sequencing GmbH, Constance, Germany.

### Sequence analysis

Paired sequencing reads were uploaded to the CLC Genomics Workbench v22, trimmed, merged, and demultiplexed using default settings. Mutation frequencies in protoplast pools at target and predicted off-target sites were determined using Amplican^35^.

## Results

### A Golden-Gate crRNA cloning system

For mutagenesis with Cas12a in plants, we determined the most efficient of three tested Cas12a orthologues and the best method for crRNA expression. In mammalian cells, three orthologues, AsCas12a, FnCas12a, and LbCas12a, were initially found capable of inducing mutations. Therefore, we compared these three orthologues' efficiencies in causing mutations in tomato cells.

**Figure 1:**
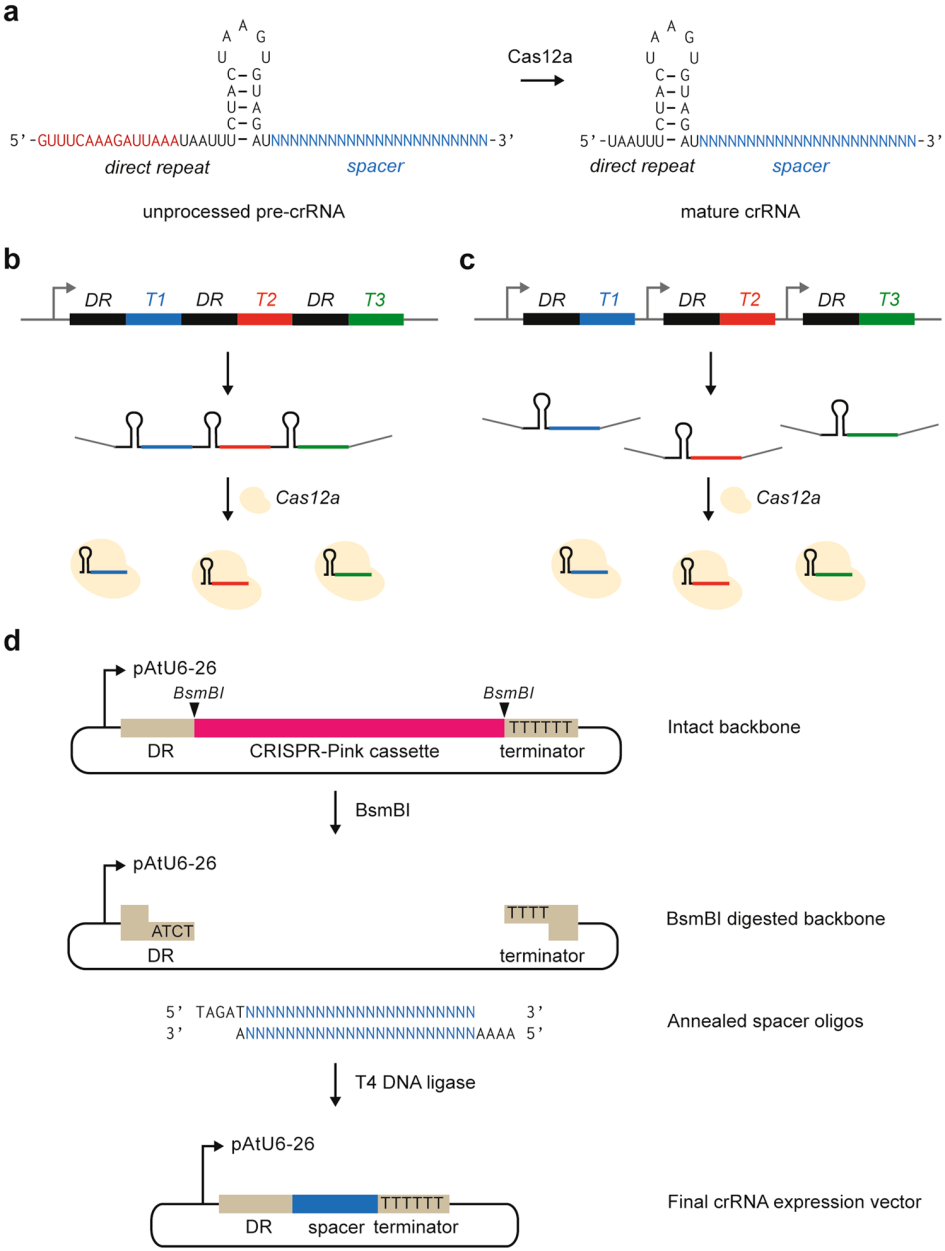
Expression of crRNAs for Cas12a. (**a**) Cas12a is capable of processing its own crRNA arrays. The sequences of the unprocessed, pre-crRNA and processed, mature crRNA for LbCas12a are shown. (**b**) and (**c**) Cas12a crRNAs can be expressed in an array (**b**), driven by a single promoter, or individually, each under the control of their own promoter (**c**). DR: direct repeat, T1-3: spacer sequences. (**d**) Cloning strategy for new crRNA expression vectors. Forward and reverse oligos encoding a new spacer are annealed and can then be cloned into the backbone vector using a *Bsm*BI-based restriction-ligation process.

As Cas12a is capable of processing its own crRNA arrays, the individual crRNAs can be expressed in two different forms: as a longer, unprocessed pre-crRNA, which still needs additional processing by Cas12a before complex formation, or the shorter, mature version, skipping the first step and facilitating direct loading of the crRNA into the Cas12a-crRNA complex (Fig. 1a). Additionally, the processing abilities of Cas12a raise the opportunity to express crRNAs as an array. In this case, multiple crRNAs are expressed in tandem as a single transcript, without additional provisions for processing as required for Cas9 sgRNAs (Fig. 1b, c).

To facilitate easy cloning of single crRNAs, each expressed by its own U6-26 promoter, we adapted Golden-Gate compatible CRISPR-Pink sgRNA cassettes (a gift from Marc Youles, The Sainsbury Laboratory). These plasmids contain an AtU6-26 promoter for sgRNA expression, followed by an operon expressing an RFP protein. This operon can easily be replaced by a spacer in a Golden Gate cut/ligate reaction, allowing pink/white screening of colonies that have successfully integrated the sgRNA. We constructed two of these plasmids for use with each of the three Cas12a orthologues: one to express the unprocessed pre-crRNA, and one for the mature crRNA. To insert new spacers into these plasmids, two oligonucleotides encoding the spacer and compatible overhangs are annealed and subsequently cloned into the plasmid (Fig. 1d, protocol in Supplementary Information 2).

### LbCas12a is the most effective orthologue for mutation induction in tomato

**Figure 2:**
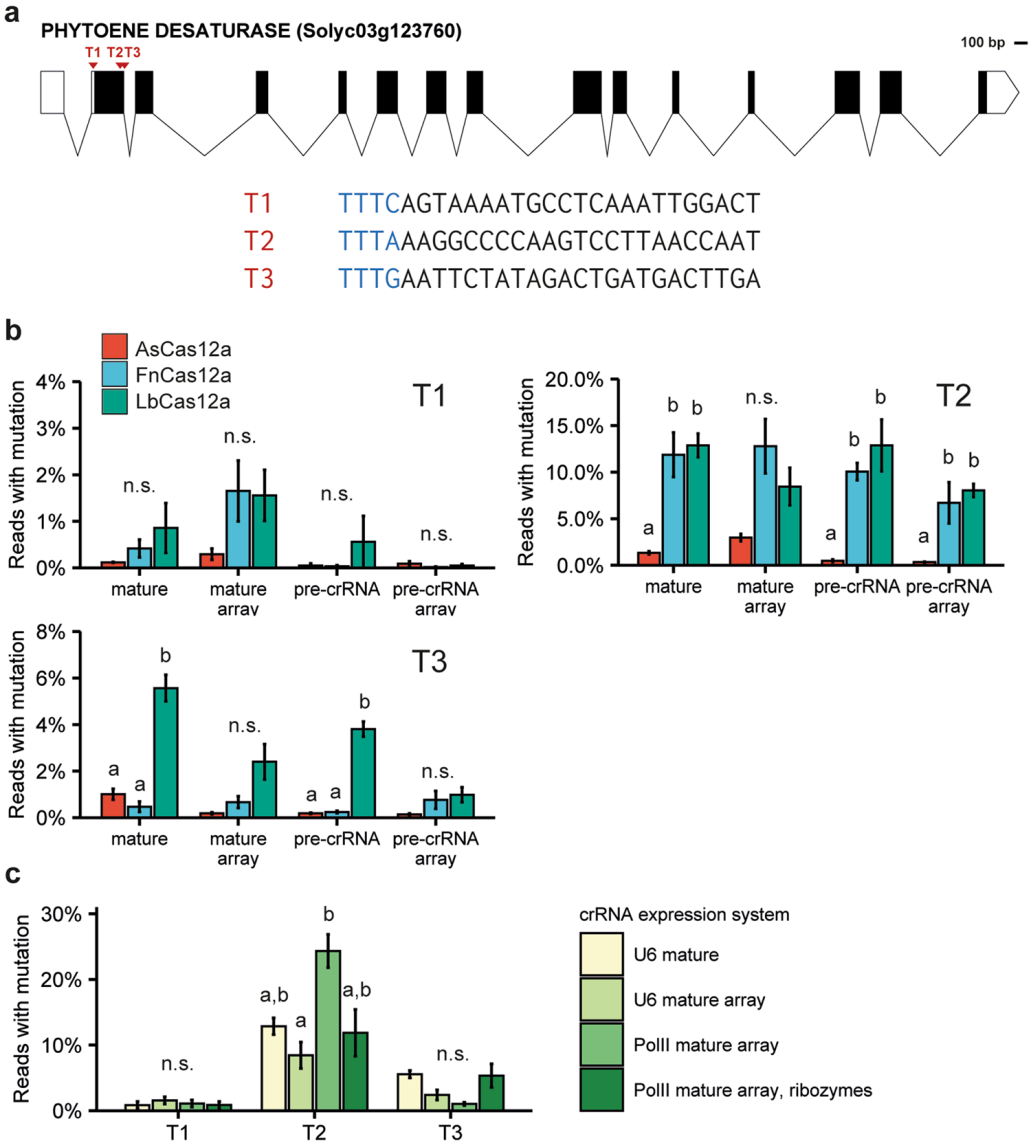
Identification of the most efficient Cas12a orthologue and method for crRNA expression. (**a**) Three target sites (T1-T3) in tomato *PHYTOENE DESATURASE (SlPDS)* were selected. (**b**) Mutation frequencies for three Cas12a orthologues and four methods of crRNA expression at three targets (T1-T3). Note the different y-axis scales for T1, T2, and T3. Error bars indicate standard error (n = 3). Different letters indicate significant differences (*p* < 0.05) between mutation frequencies induced by the different orthologues, as determined using Two-Way ANOVA followed by Tukey’s HSD Post-Hoc test. (**c**) For LbCas12a, two additional expression methods using a PolII promoter, and a PolII promoter combined with ribozymes were tested. Error bars indicate standard error (n = 3). Different letters indicate significant differences (*p* < 0.05) between mutation frequencies obtained using different crRNA expression methods, as determined using Two-Way ANOVA followed by Tukey’s HSD Post Hoc test.

For initial testing, we selected three Cas12a target sites in tomato *PHYTOENE DESATURASE (PDS) (Solyc03g123760)* (Fig. 2a). Spacers targeting these sites were cloned in the vectors for pre-crRNA and for mature crRNA expression for all three Cas12a orthologues. Each set of three level 1 vectors was then combined with the nuclease gene and a tGFP marker into a binary level 2 vector. Additionally, expression cassettes in which the three crRNAs were expressed as a single transcript from an AtU6-26 promoter—both in the pre-crRNA and mature form—were synthesized as level 1 vectors. These arrays were likewise combined with the nuclease and tGFP marker into level 2 vectors. In total, we thus made twelve level 2 vectors – each one expressing either AsCas12a, LbCas12a, or FnCas12a from a *2xCaMV35S* promoter and the three crRNAs using one of the four expression methods (see Supplementary Fig. 1a for a graphical overview). These constructs were then transfected into tomato protoplasts. The presence of the tGFP marker allowed for determining the transfection efficiency, which was similar across the three replicates and was approximately 50% (see also Fig. 4b). After the purification of DNA from the protoplast pools, the three target sites were amplified by PCR, and the resulting amplicons were subjected to next-generation amplicon sequencing. The percentage of edited reads in the pools was determined using AmpliCan (Fig. 2b)^35^.

The observed mutation frequencies varied strongly per target site and per orthologue (Fig. 2b). For target 1, none of the orthologues performed well, and mutation frequencies never reached over 2.5%. For target 2, both FnCas12a and LbCas12a performed well, whereas AsCas12a resulted in significantly lower mutation frequencies. For target 3, LbCas12a performed best, significantly outperforming AsCas12a and FnCas12a. From these results, we concluded that LbCas12a was the best choice for Cas12a mutagenesis in tomato.

For FnCas12a and LbCas12a, both the use of mature crRNAs and pre-crRNAs, either individually expressed or as an array, could result in high mutation frequencies. The highest AsCas12a mutation frequencies were obtained using mature crRNAs. For LbCas12a, individually expressed crRNAs performed slightly (but not significantly) better than their arrayed counterparts at T2 and T3. This pattern was, however, not observed at T1 or for the other orthologues (Fig. 2b). As we concluded that LbCas12a was the overall best-performing orthologue, we aimed to further test methods for crRNA expression for this orthologue.

### Several methods of crRNA expression resulted in efficient mutagenesis

It was previously reported that using a PolII promoter instead of a PolIII promoter for crRNA expression improved Cas12a editing efficiency, as did using self-cleaving ribozymes flanking the crRNA array^19,21^. As mature crRNAs generally performed comparable to or slightly better than pre-crRNAs in our previous experiment (Fig. 2b), we tested these additional expression systems only for the combination of LbCas12a and mature crRNAs (Supplementary Fig. 1b). The Cassava Vein Mosaic Virus (*CsVMV*) promoter was selected as the PolII promoter for crRNA expression. Significant differences between mutation efficiencies of the different expression systems were only found for target 2, for which the array-based crRNA expression system with the PolII promoter resulted in significantly higher mutation frequencies than the same crRNA array expression driven by the AtU6-26 promoter (Fig. 2c). As arrays or ribozymes made the system more complex but did not significantly improve mutation frequencies, we combined LbCas12a with mature, individually expressed crRNAs for Cas12a-mediated mutagenesis in subsequent experiments.

### Comparing Cas12a and Cas9 performance at overlapping target sites

**Figure 3:**
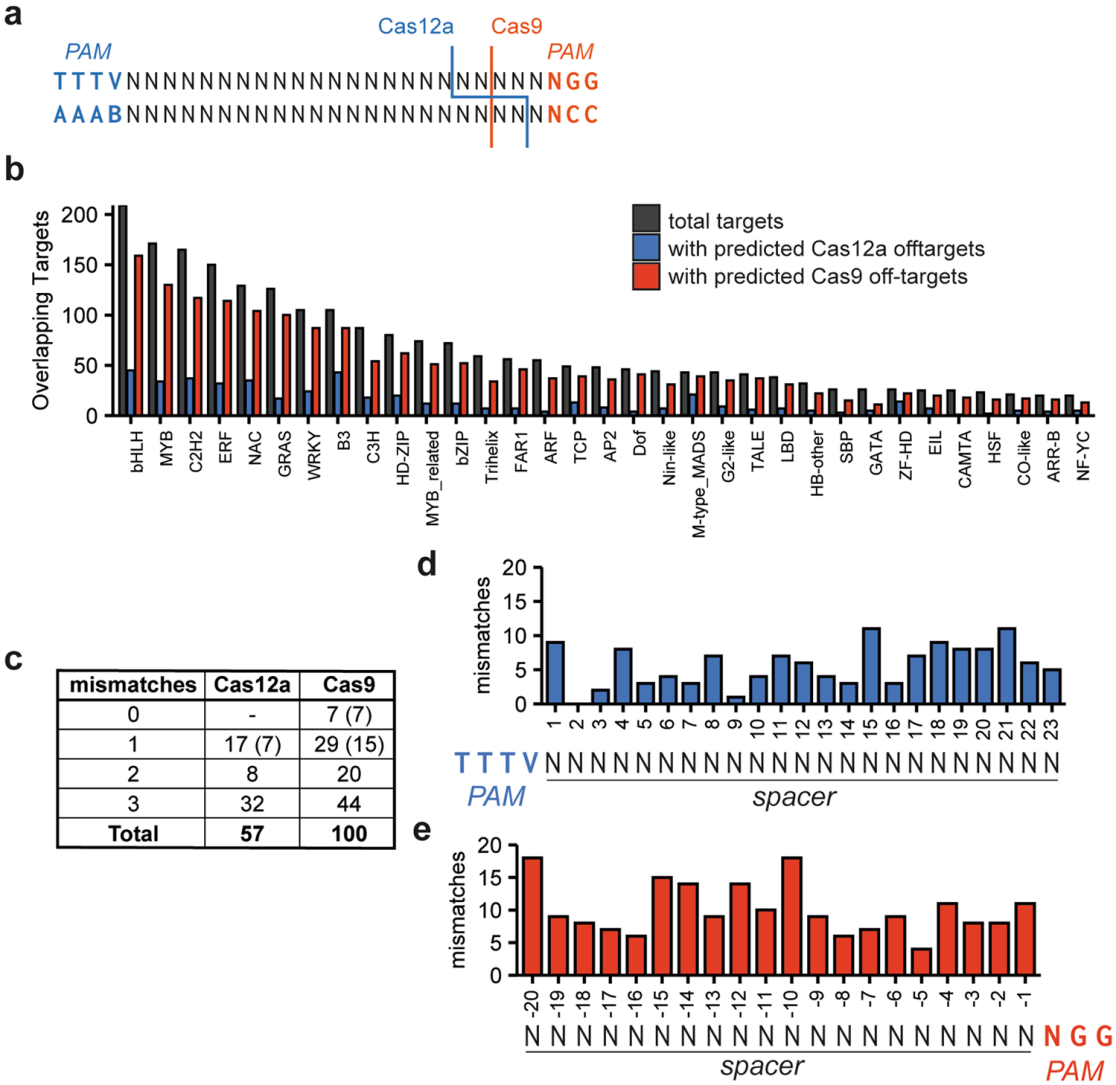
Overlapping Cas12a crRNA and Cas9 sgRNA sites for unbiased comparison of the two nucleases. (**a**) Sequence of overlapping target sites. (**b**) The number of overlapping targets found in different transcription factor families, and the number of overlapping target sites that have predicted off-target sites with a maximum of three mismatches for Cas12a and Cas9. (**c**) The number of predicted off-target sites with 0, 1, 2 or 3 mismatches for the 35 selected overlapping target sites for Cas12a and Cas9. Numbers between brackets indicate the amount of predicted off-target sites that have an insertion or deletion, leading to the formation of an RNA or DNA bulge, in addition to the number of mismatches indicated in the first column. (**d**) and (**e**) Distribution of mismatches over the length of the spacer for Cas12a (**d**) and Cas9 (**e**).

We next compared Cas9 and Cas12a efficiency, specificity, and the mutations produced. For this comparison, we identified sites where targets for Cas9 and Cas12a overlap, thus removing variation caused by differences in genomic context for the two enzymes (Fig. 3a). We identified these overlapping sites in several gene families encoding transcription factors. Using gene families allows for selecting target sites that have predicted off-target sites with a range of mismatching nucleotides. This approach provides insight into the number and position of mismatches that will enable Cas-mediated double-strand breaks and mutagenesis at off-target sites. We predicted these off-target sites with up to 3 mismatches for Cas9 and Cas12a and all identified overlapping target sites in all transcription factor families using Cas-OFFinder (Fig. 3b)^31^. In general, Cas12a target sites had fewer predicted off-target sites than Cas9 target sites, probably due to the longer spacer (23 nt for Cas12a and 20 nt for Cas9). We selected 35 overlapping target sites in the *bHLH* gene family as this family had the highest number of available overlapping targets and off-target sites (Fig. 3b). For the Cas9 sgRNAs, we selected 100 potential off-target sites with varying amounts of mismatches and, in 22 cases, an insertion or deletion compared to the target site^36^. For the Cas12a crRNAs, we selected 55 potential off-target sites, of which 7 had an additional insertion or deletion compared to the target site (Fig. 3c). Up to four potential off-target sites per target site were selected for the study. We aimed to select potential off-target sites that resulted in an as equal as possible distribution of mismatches over the length of the spacer (Fig. 3d, e). Selected target and predicted off-target sites are listed in Supplementary Dataset 1.

**Figure 4:**
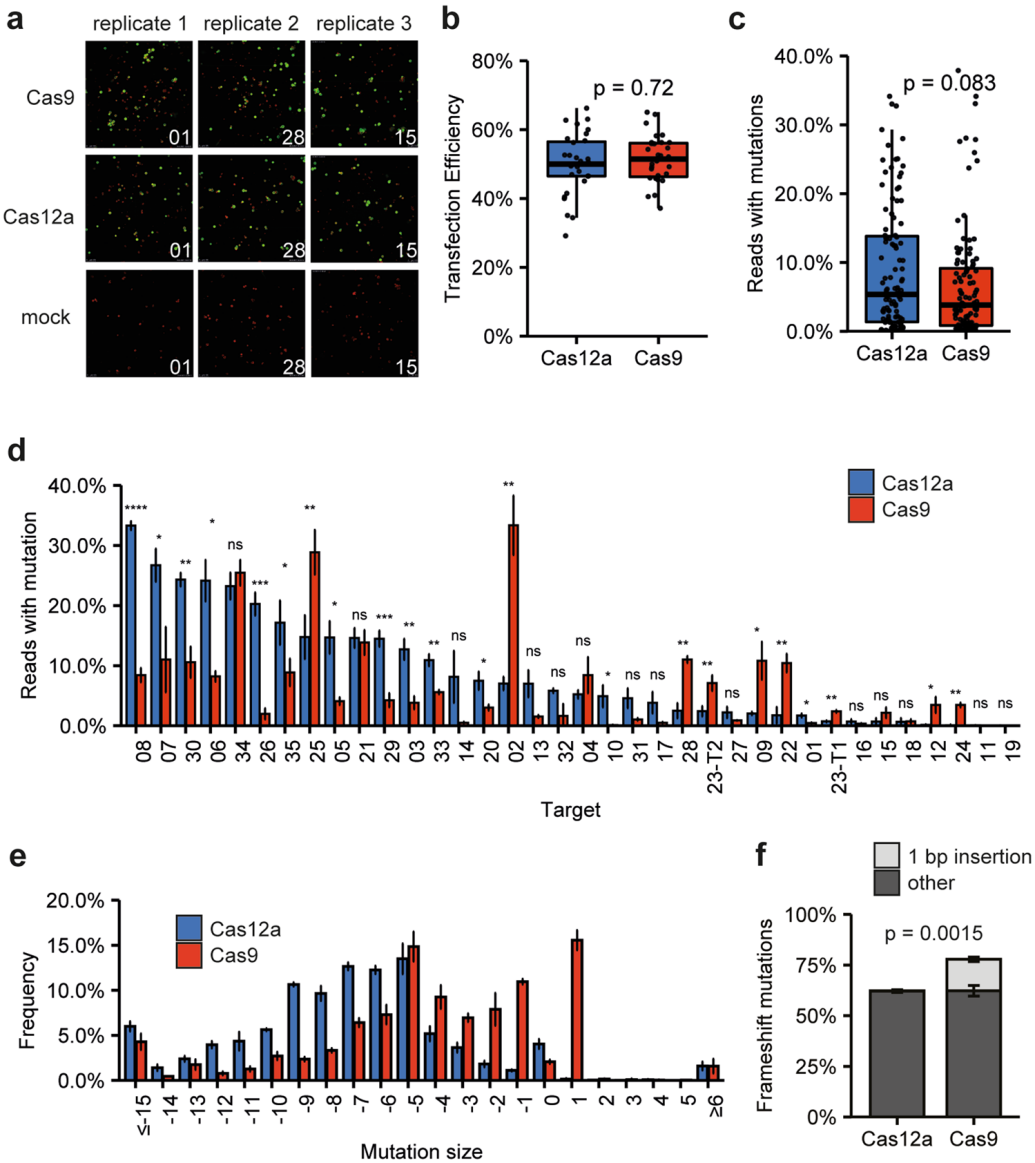
Comparison of on-target mutations for Cas12a and Cas9. (**a**) and (**b**) Comparison of transfection efficiencies of protoplasts transfected with Cas9- or Cas12a- encoding plasmids. Representative images of Cas9-, Cas12a- and mock-transfected protoplasts are shown in (**a**). Successfully transfected protoplasts show green GFP fluorescence, while untransfected protoplasts only show red autofluorescence of chloroplasts. The pictures shown belong to targets 1, 28, and 15. (**b**) Transfection efficiencies were determined by counting the number of successfully transfected protoplasts and dividing it by the total number of protoplasts. For every nuclease in every replicate, the transfection efficiency of 11 individual transfections in one 96w plate was determined. The significance of difference was determined using the Wilcoxon Test. (**c**) Comparison of mutation frequencies for Cas12a and Cas9. Every point represents the mutation frequency at a target site. The significance of differences was determined using the Wilcoxon test. (**d**) Comparison of mutation frequencies for Cas12a and Cas9 per individual target site. Error bars indicate standard deviation (n = 3). Significances were determined using Student’s T Test. Asterisks indicate significant differences (*: *p* < 0.05, **: *p* < 0.01, ***: *p* < 0.001, ****: *p* < 0.0001, ns: not significant). (**e**) Mutation pattern of Cas9 and Cas12a at target sites. Error bars indicate standard deviation (n = 3). (**f**) Percentage of total mutations that are frameshift mutations for both nucleases. Frameshift mutations resulting from 1 bp insertions are shown in light grey, all others in dark grey. Error bars indicate standard deviation (n = 3). Significant difference between the total percentage of frameshift mutations was determined by Student’s T Test.

To facilitate easy cloning of these single crRNAs or sgRNAs, we constructed level 2 vectors containing either the Cas12a or Cas9 nuclease expression cassette, a turboGFP expression cassette for monitoring transfection efficiency, and a CRISPR-Pink cassette in which the spacer can be inserted using *BsaI*-mediated restriction-ligation. Both the Cas12a crRNAs and the Cas9 sgRNAs were transcribed from an *AtU6-26* promoter in these CRISPR-Pink cassettes. For the Cas12a level 2 vector, we used an improved, *Arabidopsis-*codon optimized and thermotolerant version of LbCas12a^20^. As we had noticed that *E. coli* liquid cultures with Cas12a-containing plasmids sometimes grew poorly, we inserted an intron in *LbCas12a* to prevent expression of the Cas12a protein in *E. coli*, and did the same for Cas9. The 35 spacers were subsequently ligated in both vectors, resulting in 70 vectors. Tomato protoplasts were simultaneously transfected in a 96-well format. Transfection efficiencies were determined using confocal microscopy for Cas9- and Cas12a-transfected protoplasts and were found to be similar (Fig. 4a, b). Target site and predicted off-target site fragments were PCR-amplified, and the pooled, barcoded amplicons were sequenced.

### Cas12a and Cas9 have similar overall efficiencies but strongly different efficiencies at individual targets

We first determined and compared the on-target mutation efficiencies at every target site. In this experiment, Cas12a performed slightly – though not significantly—better overall (Fig. 4c). However, the best-performing nuclease varied per target site. Cas12a performed significantly better than Cas9 at 13 sites, and Cas9 performed better than Cas12a at 9 sites (Fig. 4d). The correlation between Cas9 and Cas12a activity at target sites was low (Supplementary Fig. 2). Previously, we correlated predicted to measured Cas9 activity and found that the so-called Azimuth score^37,38^ had some, albeit limited, predictive value^28^. Here, we calculated the DeepCpf1 score^39^ for each tested Cas12a target site and correlated this score to the obtained mutation frequency (Supplementary Fig. 3). Although DeepCpf1 could predict the top and bottom performers to some extent, the correlation was generally low.

### Cas12a induces more and larger mutations than Cas9

We compared all obtained on-target mutations for both nucleases, first by type. For Cas12a, insertions occurred at a frequency of 1.8 ± 0.4%, and deletions at 94.1 ± 0.7%. For Cas9, insertions occurred at a frequency of 17.4 ± 1.5%, of which most (89 ± 4%) were one bp, and deletions at 80.5 ± 1.6%. The distributions of mutation sizes for both enzymes are shown in Fig. 4e. Cas12a-induced deletions tend to be larger than Cas9-induced mutations. Interestingly, the characteristic peak for one bp insertions induced by Cas9 is absent for Cas12a. Likely as a result of this single difference, Cas9 caused more frameshift mutations than Cas12a (Fig. 4f).

### The frequency of off-target mutations is low for both Cas12a and Cas9

To gain more insight into the specificity of Cas12a and Cas9, we determined mutation frequencies at amplified predicted off-target sites using AmpliCan. To identify genuine Cas-induced off-target mutations as opposed to sequencing or PCR errors, we considered all off-target sites at which mutations occurred at a frequency of 0.1% of total reads or more. We disregarded any tested off-target sites for which we did not obtain a reliable wild-type control consensus sequence. Finally, we inspected the obtained mutation patterns to ensure they showed characteristics of CRISPR-induced mutations, such as insertions and deletions instead of substitutions, likely caused by PCR or sequencing errors (Supplementary Figs. 4, 5).

**Figure 5:**
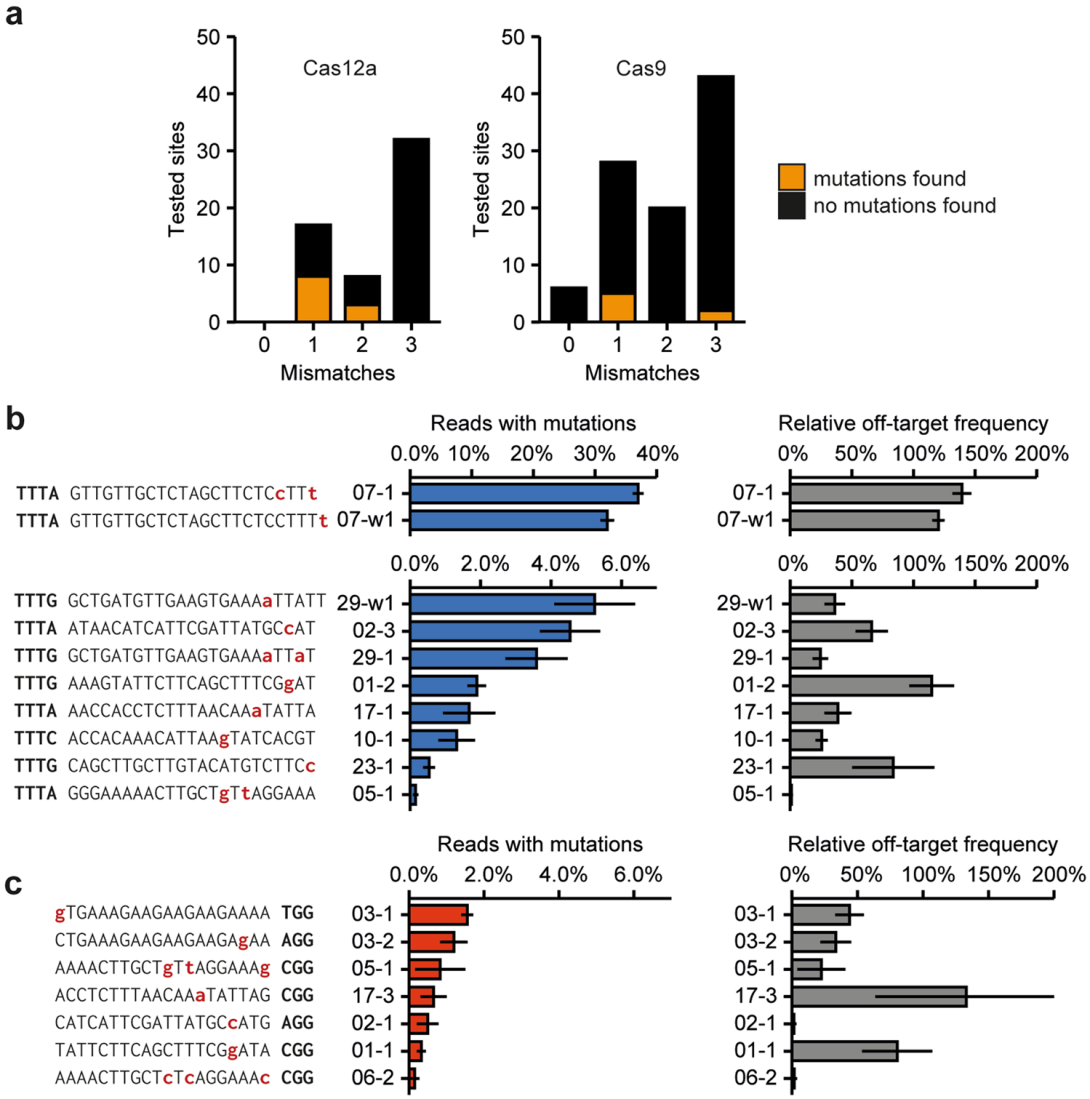
Mutated off-target sites for Cas12a and Cas9. (**a**) Overview of the number of tested off-target sites. For Cas9, the off-target sites with 0 mismatches contain an insertion or deletion leading to the formation of an RNA or DNA bulge. The number of sites at which genuine off-target mutations were identified are indicated by yellow. (**b**) and (**c**) Identified off-target sites for Cas12a (**b**) and Cas9 (**c**). Mismatches to the target site are indicated in lowercase red. Protospacer adjacent motif is indicated in bold. Mutation frequencies as well as relative off-target frequencies are shown. Relative off-target frequencies were calculated by dividing the mutation frequency at the off-target site by the mutation frequency at the target site and give a measure of the likelihood of an on- and off-target mutation occurring in the same genome. Error bars indicate standard error (n = 3).

For Cas12a, we identified 10 sites with genuine off-target mutations out of 55 tested sites; for Cas9, we identified 7 sites out of the 97 sites for which an amplicon could successfully be obtained (Fig. 5a). To estimate how often an off-target mutation and an on-target mutation would occur in the same genome, we calculated the relative off-target frequencies by dividing the off-target frequency by the on-target frequency. Figures 5b (Cas12a) and 5c (Cas9) show the absolute and relative off-target mutation frequencies.

For Cas12a, off-target mutations frequently occurred when 1 or 2 mismatches to the target occurred. However, none of these mismatches occurred in the first 14 nucleotides of the spacer. Additionally, relative off-target frequencies seem to decrease as mismatches are present closer to the PAM (Fig. 5b). No mutations were found in predicted off-target sequences with 3 mismatches.

Cas9 showed activity at sites with 1 or 3 mismatches to the target site. The off-target site with the highest mutation frequency contained only one mismatch at the position most distal from the PAM. Interestingly, mutations were also found at sites that had mismatches close to the PAM.

## Discussion and conclusion

In this study, we aimed at testing CRISPR-Cas12a mediated genome editing in tomato cells and comparing its performance to the frequently used CRISPR-Cas9. To achieve this, we first compared different Cas12a orthologues and methods for crRNA expression. We found LbCas12a to be the most efficient and robust orthologue for inducing mutations, in agreement with previous reports^17,19,40,41^. Although FnCas12a was also capable of inducing mutations at high frequency, one of the target sites used for testing (T3, Fig. 2b) that was successfully mutated by LbCas12a gave only low mutation frequencies for FnCas12a. AsCas12a performed poorly at all three tested target sites. It was shown previously that the efficiency of Cas12a-mediated genome editing, like that of Cas9-mediated genome editing, increases with temperature^42–44^. AsCas12a seemed to be more sensitive to temperature than LbCas12a^42^. Tomato protoplast experiments and tissue culture were routinely performed at 25 °C, which may be too low for AsCas12a.

As we found that LbCas12a was most efficient at mutating the tomato genome, we further investigated the best method for crRNA expression for this nuclease. Mutations could reliably be obtained with all tested crRNA expression systems, in contrast to earlier studies in rice and soybean where the use of mature crRNAs in combination with PolIII promoters resulted in no or very low mutation frequencies^18,19,41^. As the individual crRNA expression cassettes we created in this study offer greater flexibility of cloning than arrays and ribozyme-based systems, this is the method that is routinely applied in our laboratory for the construction of binary vectors for stable transformation. Although the study presented here focuses on protoplasts, our laboratory has successfully generated a large number of stably transformed tomato plants (unpublished results) with Cas12a-induced mutations using a combination of thermotolerant LbCas12a and crRNAs expressed in the mature form, cloned in the vectors as described in Fig. 1d.

To compare the performance of Cas9 and Cas12a as fairly as possible, we selected 35 overlapping target sites in the coding sequence of genes from the bHLH gene family. Overall, Cas12a showed editing at these sites at a similar level as Cas9. However, mutation rates varied strongly depending on the target site, as has been previously reported^26^. As overlapping target sites were used, characteristics such as G/C content, chromatin conformation, and epigenetic marks such as DNA methylation or histone modifications are mostly similar for the Cas12a and Cas9 target sites. However, Cas9 and Cas12a might have different preferences or tolerances for such features, affecting their efficacy. Additionally, the exact nucleosome localization might affect the availability of the target sites^45,46^.

Interestingly, some target sites showed hardly any editing for Cas9, whereas Cas12a could reliably induce mutations, such as targets 10 and 26 (Fig. 4d). For these two specific targets, Cas9 inactivity might be explained by the presence of a “TT” motif in the 3’ end of the spacer, resulting in low expression of the sgRNA^47^. This might be overcome by using pre-assembled Cas9-sgRNA complexes (RNPs) or mutated scaffold RNAs^47^.

Reliably predicting which nuclease would perform best at a specific target site before proceeding to stable transformation is desirable. Several algorithms for Cas9 exist for efficiency prediction, which are implemented in frequently used tools for sgRNA prediction, such as CRISPR-P^38,48,49^. Information for Cas12a, especially about activity in plants, is more limited. We tested the correlation between the DeepCpf1 prediction score^39^ and the obtained mutation frequencies from our dataset (Supplementary Fig. 3). crRNAs were divided into quartiles based on their DeepfCpf1 score and plotted against mutation frequencies per quartile. Although the first and fourth quartiles gave significantly lower and higher actual activities, the variation of mutation frequencies within quartiles was large, and the correlation between the DeepCpf1 score and obtained mutation frequencies was low (Supplementary Fig. 3). In the future, more high-throughput data could be obtained to specifically train algorithms to predict efficient crRNAs in plants. Apart from the obtained mutation frequencies, we also compared the mutation patterns induced by Cas9 and Cas12a at target sites. For Cas9, a significant portion of induced mutations is a one bp insertion. Previously, we and others have shown that these characteristic one bp insertions are likely often the result of the fill-in of Cas9-induced staggered DSBs with a one bp 5′ overhang, followed by subsequent ligation of the, now blunt, DNA strands^28,50–55^. LbCas12a also induces staggered cuts, with a larger 4–5 bp 5′ overhang^7^. Interestingly, no peak is found in the mutagenic spectrum for Cas12a at the + 4 and + 5 positions (Fig. 4e), indicating that the fill-in of these staggered overhangs and subsequent ligation of ends is not frequently employed for the repair of Cas12a-induced DSBs.

Cas12a-induced deletions are frequently larger than Cas9-induced deletions: for Cas9, most deletions range from 1 to 5 bp, whereas for Cas12a, most deletions range from 5 to 10 bp. It has been suggested that this difference may be caused by the fact that LbCas12a cuts distal from the PAM, outside of its “seed” sequence. As a consequence, the recognition of the target site may tolerate small mutations, and the target site may be cleaved again until a large enough deletion finally precludes recognition and cleavage^56^. An alternative explanation for the difference in mutation patterns between Cas12a and Cas9 is that a larger fraction of the Cas12a-induced mutations are caused by microhomology-mediated end-joining (MMEJ, also called alternative end joining or alt-EJ), which always induces deletions. This could either be explained by the fact that the staggered DSB caused by Cas12a preferentially triggers end-resectioning and thus MMEJ or by the fact that NHEJ-mediated repair is more often perfect as a result of the overhangs produced by Cas12a. In that case, DSBs will keep being induced until either NHEJ is unsuccessful (which only happens at low frequencies) or, more likely, the break is repaired following end-resectioning, such as in MMEJ. Either way, this bias towards end resectioning may explain why Cas12a is generally found to be more successful than Cas9 in inducing homology-directed repair (HDR)^56,57^, as end-resectioning is the first step required for this repair outcome^58–61^.

We found Cas9 to induce frameshift mutations at higher frequencies than Cas12a, which is predominantly caused by the 1 bp insertion mutations. This difference in mutation pattern may make Cas9 the more suitable option for producing knock-out mutants in protein-coding genes. Desirable phenotypes can also be obtained by tweaking the expression of genes through modification of cis-regulatory elements^62,63^. Cas12a could be the enzyme of choice for this type of genome editing because it has an A/T-rich PAM and a propensity to induce slightly larger deletions, which disrupt or delete the often short regulatory motifs present in promoters.

In studies of mammalian cells, Cas12a is generally reported to be more specific than Cas9^15,16^. Studies on Cas12a-induced off-target mutations in plants have so far been conducted on a small scale^64–66^ but likewise indicate that Cas12a does not frequently induce off-target mutations. To acquire more data on Cas12a specificity, we selected 57 predicted off-target sites with 1–3 mismatches to the spacer and investigated them for the presence of off-target mutations. At 10 out of 57 sites, off-target mutations were identified. For Cas9, we investigated 100 predicted off-target sites and found evidence of off-target mutation at seven sites. Cas12a off-target activity was strongly linked to mismatches at the 3’ end of the spacer, distal to the so-called “seed sequence”. Conversely, Cas9 off-target sites with mismatches proximal to the PAM were still found to be mutagenized, albeit at low frequencies (Fig. 5c)^67^. Based on these results, the existence of a seed sequence may be more applicable to Cas12a than Cas9. This would make potential high-risk Cas12a off-target sites easier to predict and, therefore, to avoid.

In this study, we selected spacers with a length of 23 nucleotides. However, previous research has shown that spacers as short as 19 nucleotides retain almost complete activity^7,14^. It is therefore not surprising that the four nucleotides most distal to the PAM add little to nothing to editing specificity, resulting in high relative off-target frequencies at these sites (Fig. 5b).

Concluding, we have shown that LbCas12a can reliably and specifically induce mutations in the tomato genome. Our high-throughput testing methods allowed us to assess Cas12a orthologues, crRNA expression systems, efficiency at target sites, and specificity. Together with constructing a convenient, Golden Gate-compatible cloning system for crRNAs, this work helps lay the foundation for routine application of Cas12a to induce mutations in the tomato genome.

### Supplementary Information

Supplementary Information 1.

Supplementary Information 2.

Supplementary Information 3.

Supplementary Information 4.

## Data Availability

All raw sequencing data are available from NCBI-SRA, BioProject accession number PRJNA980545.
