# Supplementary material for: Comparison of Cas12a and Cas9-mediated mutagenesis in tomato cells
Source: Sci Rep. 2024 Feb 24;14:4508. doi: 10.1038/s41598-024-55088-4 (PMC10894265; doi:10.1038/s41598-024-55088-4)
Supplement: Supplementary file 1 — Supplementary Information 1. [file 41598_2024_55088_MOESM1_ESM.pdf]

Overview

In this document:

Supplementary Information 1 – Sequences of arrays synthesized by GenScript for crRNA expression

Supplementary Information 2 - Protocol for cloning new spacers in CRISPR-pink-based crRNA backbones

Supplementary Figure 1 – Schematic overview of plasmids used in this study

Supplementary Figure 2 - Pearson correlation of Cas12a and Cas9 mutation frequencies

Supplementary Figure 3 - Correlation of predicted and measured activity of Cas12a crRNAs

Supplementary Figure 4 – Variation plots of identified Cas12a off-target sites

Supplementary Figure 5 – Variation plots of identified Cas9 off-target sites

Provided separately:

Supplementary Dataset 1 – Overview of all selected overlapping target sites and selected off-target sites

Supplementary Dataset 2 – Oligonucleotide sequences for cloning crRNAs and sgRNAs in expression vectors

Supplementary Dataset 3 – Primers used in this study

Supplementary Information 1 - Sequences of arrays synthesized by GenScript for crRNA expression

In all sequences:

yellow : BpiI recognition and cut sites
turquoise: AtU6-26 promoter
red : direct repeat
gray : spacer

(1/6) Array for AsCas12a, mature crRNAs

gaagacaattacttcaaaagtcccacatcgcttagataagaaaacgaagctgagtttatatacagcta
gagtcgaagtagtgattGTAATTTCTACTCTTGTAGATTGCCACTCGTTTAACTTCTGAGGTAATTTC
TACTCTTGTAGATAGTAAAATGCCTCAAATTGGACTTAATTTCTACTCTTGTAGATAAGGCCCCAAGT
CCTTAACCAATTAATTTCTACTCTTGTAGATAATTCTATAGACTGATGACTTGAttttttttcagatt
gtcttc

(2/6) Array for AsCas12a, precrRNAs

gaagacaattacttcaaaagtcccacatcgcttagataagaaaacgaagctgagtttatatacagcta
gagtcgaagtagtgattGTCAAAAGACCTTTTTAATTTCTACTCTTGTAGATTGCCACTCGTTTAACT
TCTGAGGGTCAAAAGACCTTTTTAATTTCTACTCTTGTAGATAGTAAAATGCCTCAAATTGGACTGTC
AAAAGACCTTTTTAATTTCTACTCTTGTAGATAAGGCCCCAAGTCCTTAACCAATGTCAAAAGACCTT
TTTAATTTCTACTCTTGTAGATAATTCTATAGACTGATGACTTGAttttttttcagattgtcttc

(3/6) Array for FnCas12a, mature crRNAs

gaagacaattacttcaaaagtcccacatcgcttagataagaaaacgaagctgagtttatatacagcta
gagtcgaagtagtgattGTAATTTCTACTGTTGTAGATTGCCACTCGTTTAACTTCTGAGGTAATTTC
TACTGTTGTAGATAGTAAAATGCCTCAAATTGGACTTAATTTCTACTGTTGTAGATAAGGCCCCAAGT
CCTTAACCAATTAATTTCTACTGTTGTAGATAATTCTATAGACTGATGACTTGAttttttttcagatt
gtcttc

(4/6) Array for FnCas12a, precrRNAs

gaagacaattacttcaaaagtcccacatcgcttagataagaaaacgaagctgagtttatatacagcta
gagtcgaagtagtgattGTCTAAGAACTTTAAATAATTTCTACTGTTGTAGATTGCCACTCGTTTAAC
TTCTGAGGGTCTAAGAACTTTAAATAATTTCTACTGTTGTAGATAGTAAAATGCCTCAAATTGGACTG
TCTAAGAACTTTAAATAATTTCTACTGTTGTAGATAAGGCCCCAAGTCCTTAACCAATGTCTAAGAAC
TTTAAATAATTTCTACTGTTGTAGATAATTCTATAGACTGATGACTTGAttttttttcagattgtctt
c

(5/6) Array for LbCas12a, mature crRNAs

gaagacaattacttcaaaagtcccacatcgcttagataagaaaacgaagctgagtttatatacagcta
gagtcgaagtagtgattGTAATTTCTACTAAGTGTAGATTGCCACTCGTTTAACTTCTGAGGTAATTT
CTACTAAGTGTAGATAGTAAAATGCCTCAAATTGGACTTAATTTCTACTAAGTGTAGATAAGGCCCCA
AGTCCTTAACCAATTAATTTCTACTAAGTGTAGATAATTCTATAGACTGATGACTTGAttttttttca
gattgtcttc

(6/6) Array for LbCas12a, precrRNAs

gaagacaattacttcaaaagtcccacatcgcttagataagaaaacgaagctgagtttatatacagcta
gagtcgaagtagtgattGTTTCAAAGATTAAATAATTTCTACTAAGTGTAGATTGCCACTCGTTTAAC
TTCTGAGGGTTTCAAAGATTAAATAATTTCTACTAAGTGTAGATAGTAAAATGCCTCAAATTGGACTG
TTTCAAAGATTAAATAATTTCTACTAAGTGTAGATAAGGCCCCAAGTCCTTAACCAATGTTTCAAAGA
TTAAATAATTTCTACTAAGTGTAGATAATTCTATAGACTGATGACTTGAttttttttcagattgtctt
c

Supplementary Information 2 – Protocol for cloning new spacers in crRNA backbones

Step 1. Ordering oligos

Once you've selected your 23 nt target (websites such as http://crispor.tefor.net/ can help you with this), you can order oligos. You need two oligos: one forward, one reverse. To create the correct overhangs, attach 5'-TAGAT-3' at the 5' end of your forward oligo. Attach 5'-AAAA-3' to the 5' end of your reverse oligo and 5'-A-3' to the 3' end of your reverse oligo. The T/A pair (indicated in red below) is needed because the nucleotide was accidentally omitted from the LbCas12a direct repeat sequence while cloning the backbone vectors. It is necessary for proper function, so please make sure to include it.

Example:

You selected the following target (including PAM, underlined here): 5'-TTTAAAGGCCCCAAGTCCTTAACCAAT-3'

Your forward oligo will be: 5'-TAGATTAAGGCCCCAAGTCCTTAACCAAT-3'.

Your reverse oligo will be: 5'-AAAAATTGGTTAAGGACTTGGGGCCTTA-3'

Step 2. Annealing oligos

Annealing oligos can be performed in multiple ways. This is one method:

1. Dilute your oligo's to 10 mM
2. To a PCR tube, add:

Ingredient	Amount
FW oligo (10mM)	2 uL
REV oligo (10mM)	2 uL
Annealing buffer*/PCR buffer (10x)	2 uL
MilliQ to 20 uL	14 uL

* 10x annealing buffer: 100mM Tris pH 7.5, 10 mM EDTA, 500 mM NaCl. You can also use a PCR buffer.

3. Mix well, and in a thermocycler, cook the mix and slowly let it cool down to room temperature:

95 degrees - 5 minutes
90 degrees - 1 minute
go back to previous step 40x, each time decrease the temperature 1.5 degrees (=cool to room temperature)
hold forever at 12 degrees

A lot of variations to the abovementioned thermocycler protocol will work.

You can even make your mix in a 1.5 mL Eppendorf tube, cook for ~5 min at 95 degrees in a heat block, take it out and let slowly cool to room temperature on a lab bench (~20 min).

Step 3. Restriction/Ligation and transformation to E. coli

Pipette & mix together in a PCR tube:

Ingredient	Amount
Cas12aPINK backbone vector (100-200ng)	1 uL
Ligated oligo	2 uL
Buffer Tango*	2 uL
ATP (10 mM)	2 uL
BsmBI*	1 uL
T4 DNA ligase	1 uL
MilliQ to 20 uL	11 uL

* BsmBI is available from different manufacturers. Check which one you have and use the corresponding buffer. The BsmBI (Esp3I) available from ThermoFisher, which cuts optimally in Tango buffer, is very efficient as it cuts at 37 degrees. The BsmBI-v2 available from NEB, which cuts optimally in Buffer 3.1, is less efficient as it has a temperature optimum at 55 degrees (but do NOT use 55 degrees in your restriction/ligation protocol, as it will inactivate the T4 ligase!). This one can still be used, but it might be advisable to plate a bit more of your E. coli transformation.

Perform 10 cycles of restriction/ligation in a thermocycler, followed by a final restriction and heat inactivation steps:

37 degrees - 5 minutes (10x)
16 degrees - 5 minutes (10x)
50 degrees - 10 minutes
80 degrees - 10 minutes
hold at 12 degrees forever

Transform to E. coli (we usually transform 2 uL to 50 uL of electrocompetent E. coli). Plate on LB plates with added carbenicillin (or ampicillin; carbenicillin prevents the formation of satellite colonies). White colonies are correct; pink colonies have not successfully taken up the spacer.

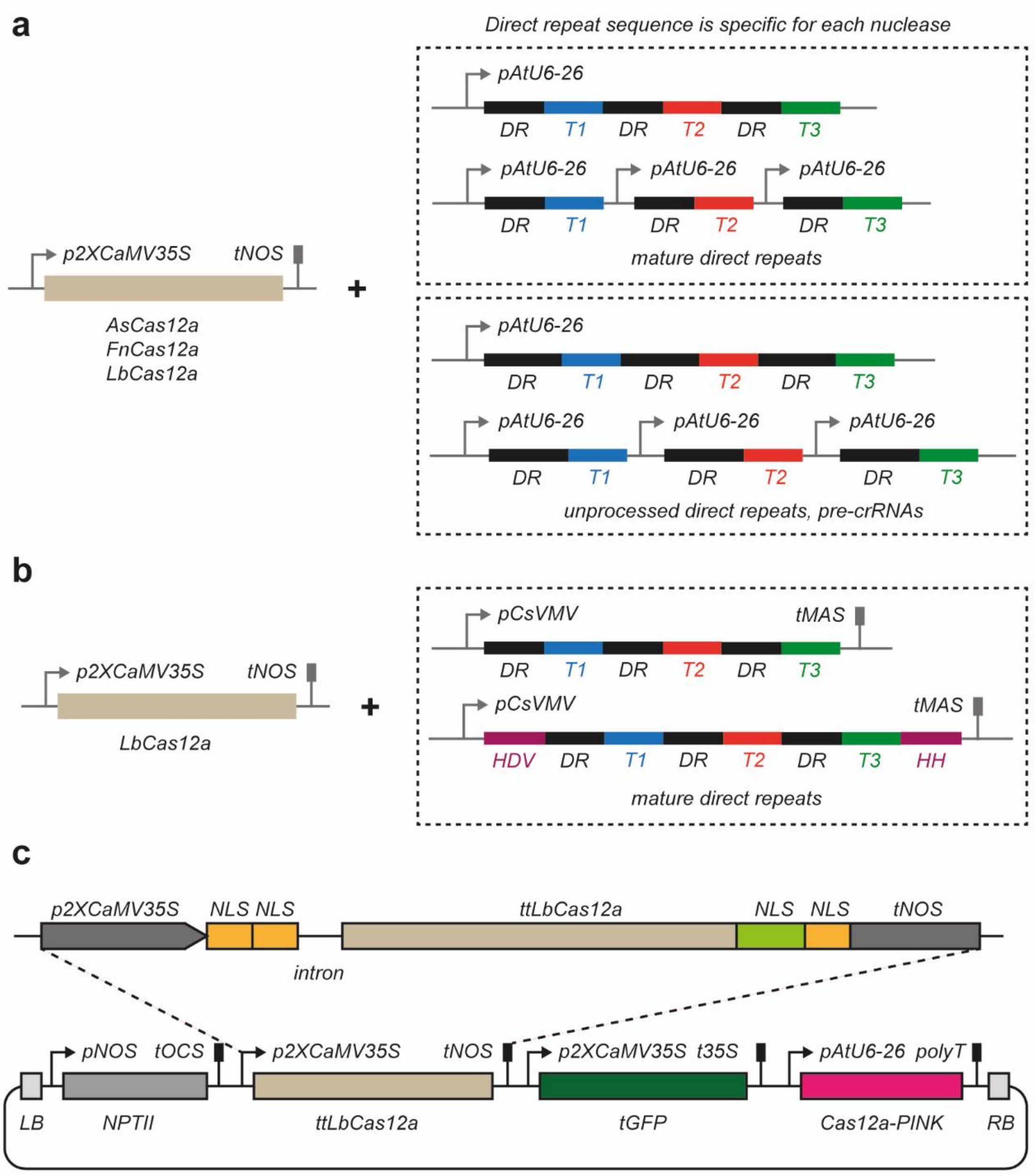

Supplementary Figure 1 – Schematic overview of plasmids used in this study. DR: Direct Repeat, T1-T3: spacers for T1-T3. (a) Plasmids used to identify the most efficient Cas12a orthologue and method for crRNA expression. Each Cas12a orthologue (*AsCas12a*, *FnCas12a* and *LbCas12a*) was tested with four methods of crRNA expression. Either the mature, or the unprocessed pre-crRNA form of the direct repeat was used. The crRNAs were either expressed together as an array, or each separately by an AtU6-26 promoter. Twelve plasmids were constructed in total. (b) For LbCas12a, two additional methods of crRNA expression were tested: an array expressed by the PolII CsVMV promoter, and an array expressed by the PolII CsVMV promoter and flanked by ribozymes. HDV: Hepatitis Delta Virus ribozyme, HH: hammerhead ribozyme. (c) Binary level 2 vector used for direct cloning of a single crRNA. In this vector, the thermotolerant version of LbCas12a was used (Schindele and Puchta, 2020). To this version, two extra SV40 nuclear localization sites (NLS, yellow) were added at the 5' end, together with a potato IV2 intron. At the 3' end, there is one nucleoplasmin NLS (green) and another SV40 (yellow) NLS. Single crRNA spacers can be cloned in the Cas12a-PINK cassette using *Bsa*I, as *NPTII* contains a *BsmB*I site. This is different from the level 1 Cas12a-PINK cassettes, where *BsmB*I is used.

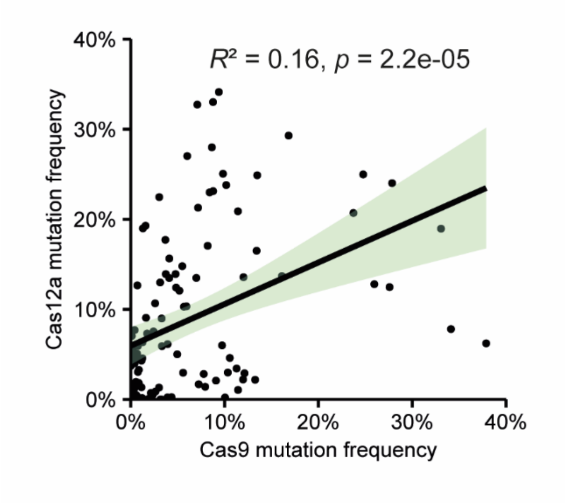

Supplementary Figure 2 – Pearson correlation of Cas12a and Cas9 mutation frequencies. The green shaded area indicates the 95% confidence interval.

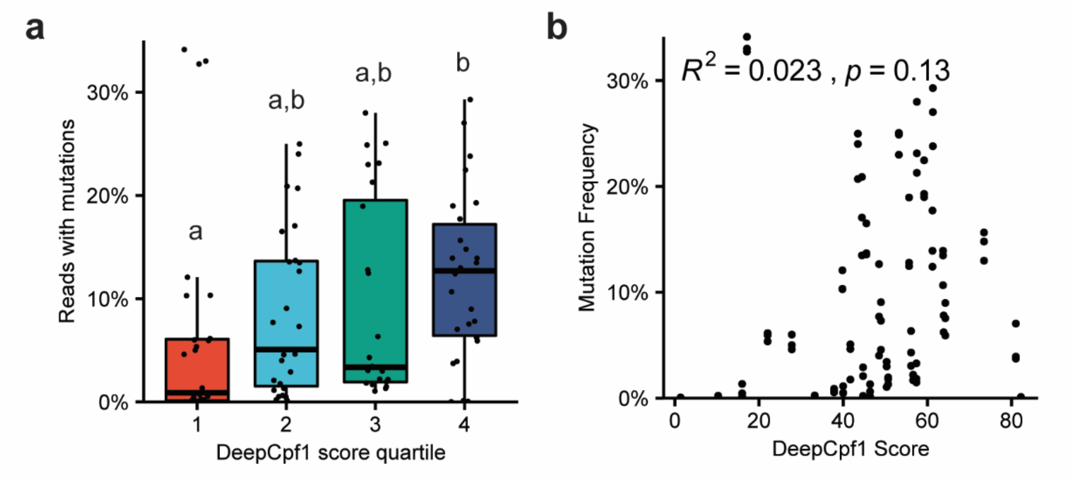

Supplementary Figure 3 – Correlation of predicted and measured activity of Cas12a crRNAs. (a) The crRNAs were divided in quartiles based on their DeepCpf1 score. The horizontal line in the box plots indicates the median, boxes represent the 2nd and 3rd quartile, and bottom and top whiskers indicate the 1st and 4th quartile, respectively. Significant differences between mutation frequencies of sgRNAs in each quartile were determined by Kruskal-Willis test, followed by Wilcoxon's Rank Sum test for pairwise comparison. Different letters indicate significant differences between groups ($p < 0.05$). (b) Scatterplot showing the distribution and Pearson correlation of DeepCpf1 score and obtained mutation frequencies.

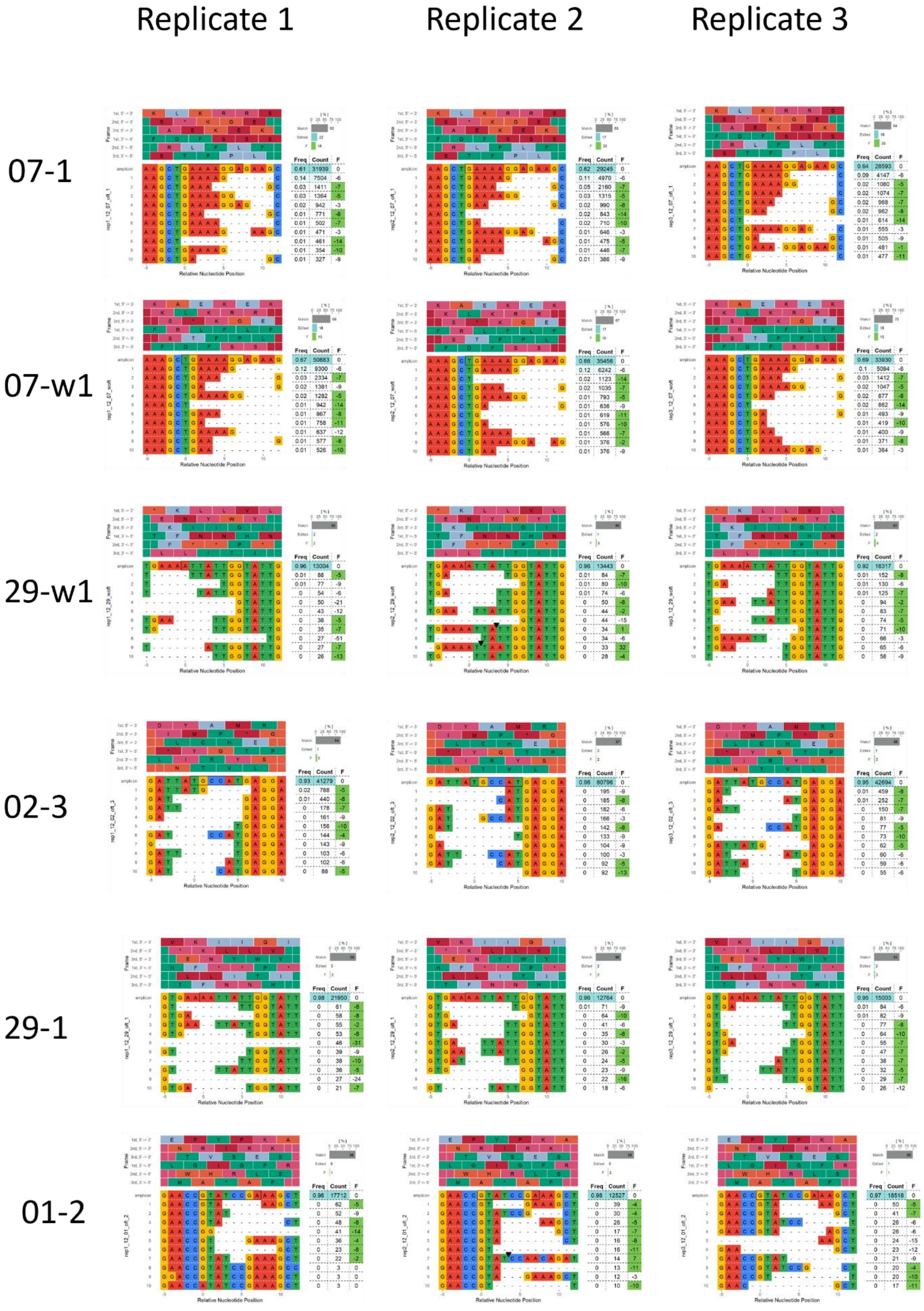

(Figure continues on next page)

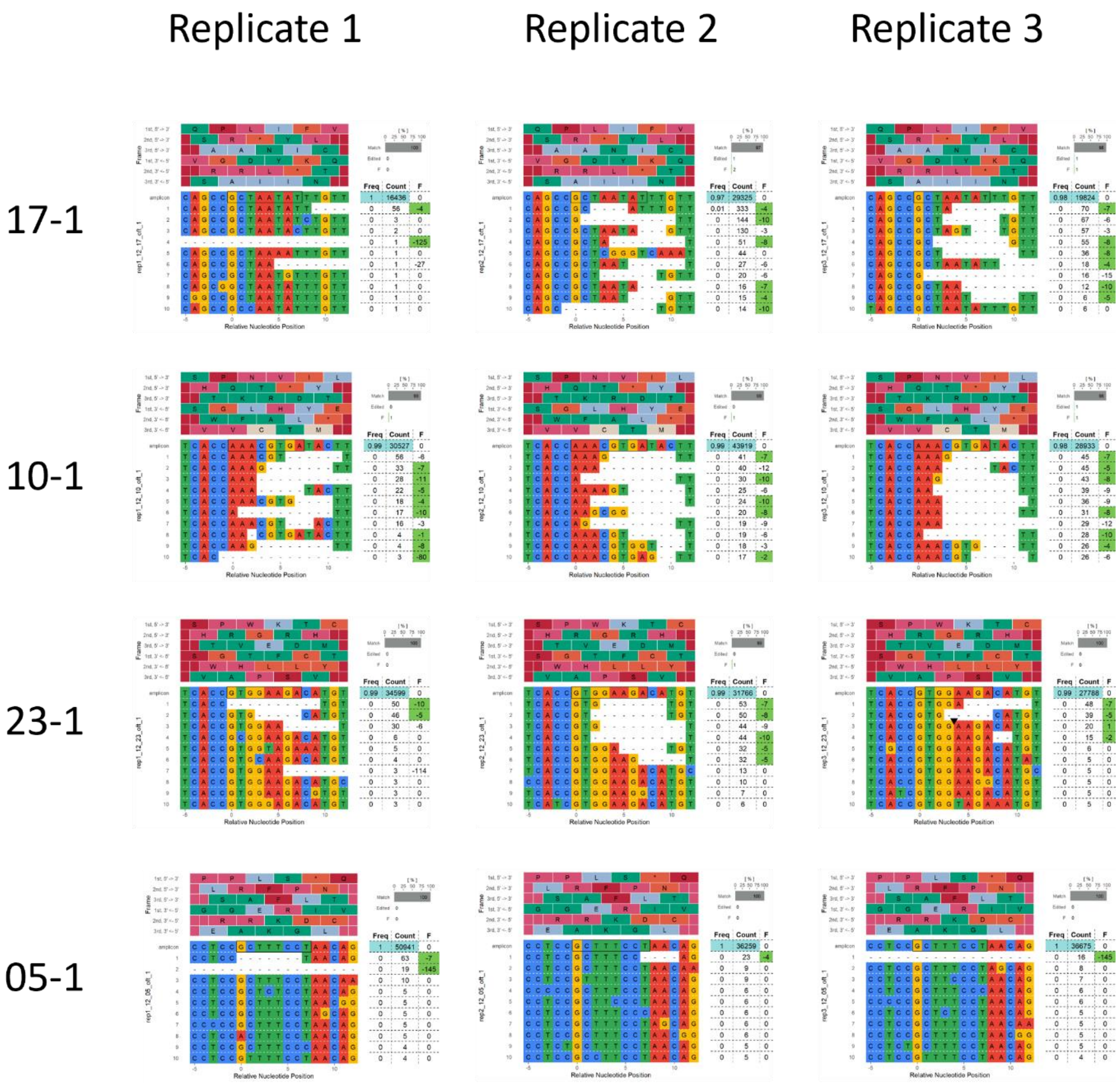

Supplementary Figure 4 – Variation plots of identified Cas12a off-target sites. The identifier on the left corresponds to the tested off-target site as described in Supplementary Dataset 1. Three biological replicates are shown per off-target site. Every plot shows all possible reading frames (top left), a bar chart depicting the percentage of reads matching the wildtype sequence (Match), percentage of edited reads (Edit) and percentage of edited reads with a frameshift mutation (F) (top right), the reference amplicon sequence around the predicted Cas12a cut site and the ten most frequently occurring alleles (bottom left), together with their frequencies (Freq), read counts (Count) and the size of the insertion or deletion (F) (bottom right).

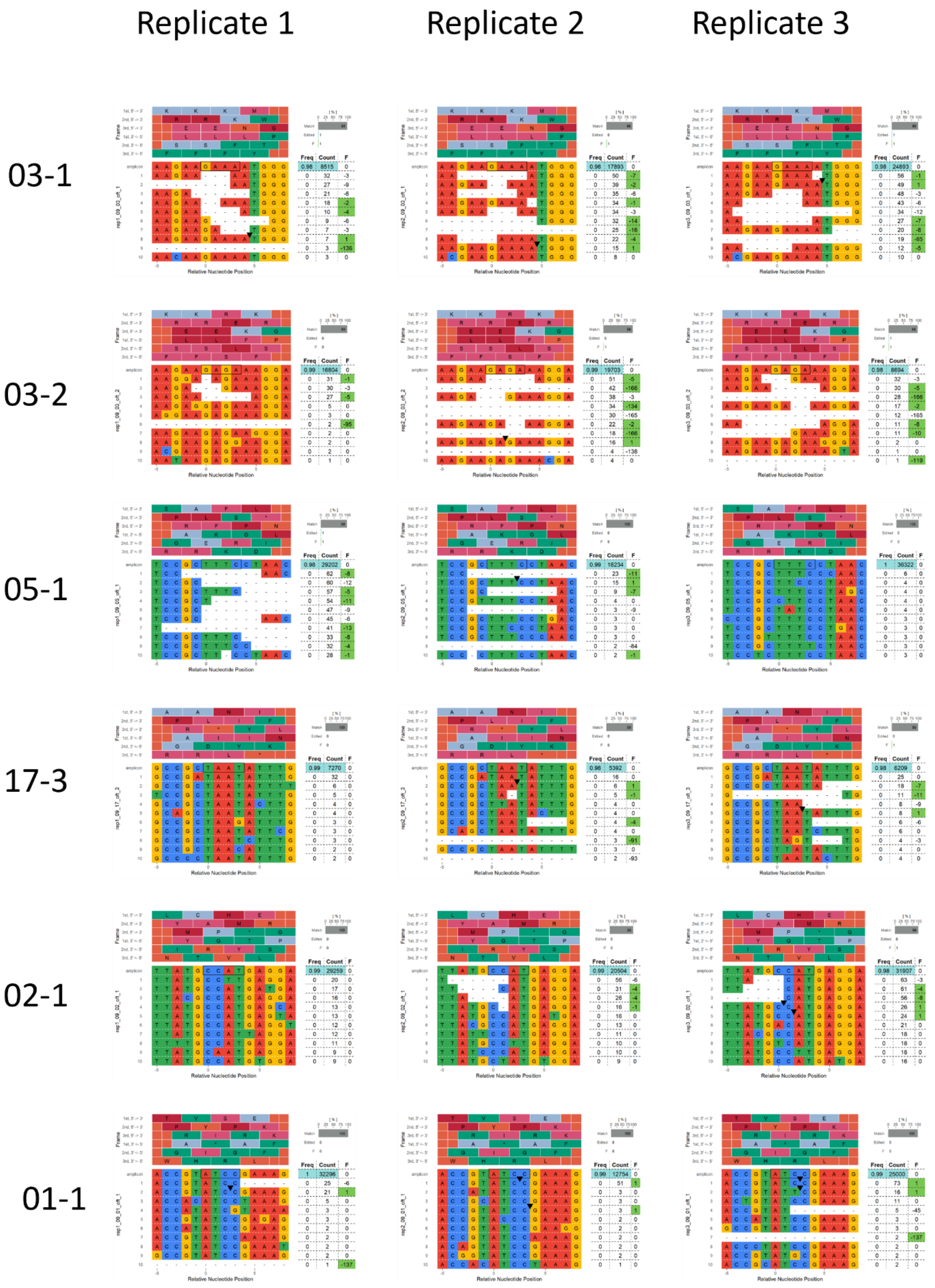

(Figure continues on next page)

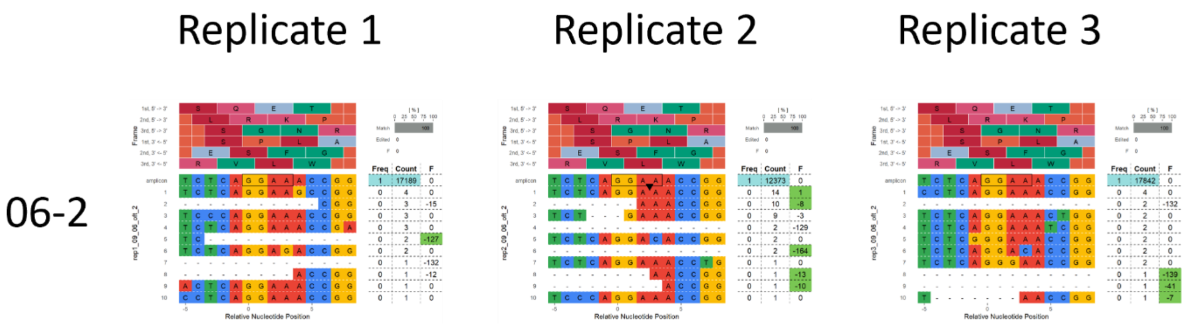

Supplementary Figure 5 – Variation plots of identified Cas9 off-target sites. The identifier on the left corresponds to the tested off-target site as described in Supplementary Dataset 1. Three biological replicates are shown per off-target site. Every plot shows all possible reading frames (top left), a bar chart depicting the percentage of reads matching the wildtype sequence (Match), percentage of edited reads (Edit) and percentage of edited reads with a frameshift mutation (F) (top right), the reference amplicon sequence around the predicted Cas9 cut site and the ten most frequently occurring alleles (bottom left), together with their frequencies (Freq), read counts (Count) and the size of the insertion or deletion (F) (bottom right).
